# Dietary acid load and esophageal cancer risk: A case‐control study

**DOI:** 10.1111/1759-7714.14612

**Published:** 2022-08-21

**Authors:** Alvaro Luis Ronco, Wilner Martínez‐López, Juan M. Calderón, Maximilian Andreas Storz

**Affiliations:** ^1^ Unit of Oncology and Radiotherapy Pereira Rossell Women's Hospital Montevideo Uruguay; ^2^ Academic Unit on Radiation Protection, Faculty of Medicine University of the Republic Montevideo Uruguay; ^3^ Biomedical Sciences Center University of Montevideo Montevideo Uruguay; ^4^ Department of Internal Medicine II Center for Complementary Medicine, Freiburg Medical Center – Faculty of Medicine, University of Freiburg Freiburg Germany

**Keywords:** cancer, dietary acid load, epidemiology, PRAL, esophagus, NEAP

## Abstract

**Background:**

A high dietary acid load (DAL) can produce metabolic acidosis, which is linked to cancer development through mechanisms of inflammation and cell transformation. There is limited epidemiological evidence linking DAL and cancer risk; however, none of the published studies focused on DAL and esophageal cancer (EC) risk in particular. Therefore, we sought to explore this association in the present study.

**Methods:**

A case‐control study was performed in 1295 male patients (185 squamous cell EC cases and 1110 age‐frequency and urban/rural residence matched controls) through a multitopic inquiry, including a food frequency questionnaire. Food‐derived nutrients were calculated from available databases. The DAL was calculated based on two validated measures: Potential renal acid load (PRAL) score and net endogenous acid production (NEAP) score. Odds ratios (OR) and their 95% confidence intervals (95% CI) were estimated by unconditional logistic regression, adjusting for confounders.

**Results:**

We found direct, significant associations between dietary acid load and EC risk: (OR = 2.28, 95% CI: 1.44–3.61, ptrend <0.0001) and (OR = 2.17, 95% CI: 1.38–3.41, ptrend <0.0001) for highest PRAL and NEAP tertiles, respectively. Our data raise the possibility that a high DAL may contribute to EC development. Both acid load scores were directly associated with animal‐based foods (mainly meat) and inversely associated with the intake of plant‐based foods.

**Conclusion:**

To the best of our knowledge, this is the first epidemiological case–control study analyzing associations of DAL and squamous cell EC risk. Further research is warranted to confirm our findings.

## Graphical abstract

Study summary. Modified from Servier Medical Art database by Servier (Creative Commons 3.0).

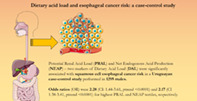

## INTRODUCTION

Esophageal cancer (EC) is the eighth most common cancer type worldwide and accounts for approximately 500 000 global cancer deaths annually.^1^, ^2^ While the world's age‐standardized incidence rate is 9.3/10^5^ people, there is a 12‐fold ratio when comparing the highest rates in Eastern Asia (12.2/10^5^) and the lowest rates in Central America (0.98/10^5^). Interestingly, analyzed by country, the extreme rates belong to Africa: the highest is in Malawi (18.7/10^5^), whereas the lowest is in Guinea (0.42/10^5^), making the ratio higher than 40:1.^2^

It has the potential to metastasize at early stages and is characterized by disproportionally high mortality rates and a poor prognosis.^2^, ^3^ EC occurs more often in males than in females and becomes more common with older age.^2^, ^4^ Furthermore, among American inhabitants, men have around four‐fold higher death rates.^5^ However, different survival analysis models have not shown differences between men and women.^4^ Recent studies suggested that almost 90% of cases occur in individuals aged 55 years or older.^1^, ^2^

The two most common histological cancer types include esophageal adenocarcinoma (EAC) and esophageal squamous cell carcinoma (ESCC),^6^ which usually occur in either the proximal (ESCC) or distal (EAC) esophagus, respectively.^7^ The global prevalence of both subtypes is in transition; and although ESCC remains the most prevalent type worldwide, EAC is quickly becoming the most common type in developed countries.^2^, ^6^

In addition, tobacco smoking, alcohol drinking, and very hot “mate” drinking are already reported risk factors for ESCC.^8^, ^9^, ^10^, ^11^, ^12^ This malignancy has also been thoroughly studied from the nutritional viewpoint in the Uruguayan population.^13^, ^14^, ^15^, ^16^

There are several established risk factors for EC that may also affect patient prognosis and survival. It is now widely accepted that EC is often preceded by chronic local inflammation in the esophagus which may alter normal cell signaling and growth.^2^ Illustrative examples include regular alcohol abuse as a risk factor for ESCC and Barret esophagus (BE) as a risk factor for EAC.^17^, ^18^ Tobacco use increases the risk for both cancer types.^19^

Of note, there is now increasing evidence highlighting the pivotal role of nutritional risk factor for esophageal cancer risk and prognosis.^20^ A high intake of meat, hot foods and beverages, and salty foods may increase the risk of EC, whereas an increased consumption of plant foods may decrease the risk of EC.^21^, ^22^ A high intake of vegetables and fruits may be of particular benefit in this context,^23^ because both are abundant in folate, vitamin C, and polyphenols which may exert favorable effects on EC risk.^24^, ^25^

The composition of diet may also influence the body's acid–base balance^26^ and a regular consumption of acidogenic dietary components may increase dietary acid load (DAL), which has been closely linked to chronic low‐grade inflammation and subsequent cell transformation.^26^, ^27^ Chronic tissue inflammation may thus stimulate carcinogenesis or tumor progression;^28^, ^29^ and adherence to an acidogenic diet has been associated with an increased risk for some types of cancers.^30^ Two recently published independent meta‐analyses revealed positive associations of a high DAL and a higher risk of cancer incidence.^30^, ^31^

A high DAL has been associated with the following malignancies in large epidemiological studies on cancer: prostate,^32^ bladder,^33^ breast,^34^, ^35^ lung,^36^ colorectum,^37^ head and neck,^38^ and pancreatic cancer.^39^ The reservation must be made that investigations in this particular field are still scarce, and whether a high DAL may increase the risk for EC has not yet been examined in particular.

In light of the conceivable pathomechanisms that may link several cancers (particularly low‐grade chronic inflammation as a common risk factor for ESCC), we sought to address this gap in the literature. To investigate the potential association between an acidogenic diet and EC, we performed a case‐control study in Montevideo, Uruguay.

## METHODS

### General information

This secondary data analysis uses data from a large Uruguayan multisite case‐control study investigating potential associations between environmental factors and the risk of cancer in the country's capital, Montevideo. The methods have been described previously in great detail^32^ A brief summary of the main study characteristics and case selection is given below. Data for this case‐control study was gathered between 1996 and 2004. All newly diagnosed cases of ESCC in men registered in Uruguay's capital were considered eligible for this study. Trained study personnel who were not aware of the main research goals performed routine screenings of current hospital records to identify potentially eligible participants at Montevideo's four largest hospitals. Hospital sites included Hospital Maciel, Hospital Pasteur, Hospital de Clinicas, and the National Oncology Institute. The study team contacted all potential study participants (including those suffering from esophageal cancer and potential controls) and invited them to participate in face‐to‐face interviews. Proxy interviews were generally not accepted.

### Selection of cases and controls

This secondary data analysis is based on a sample that comprised *n* = 1295 individuals. We identified a total of *n* = 185 cases of esophageal cancer, which were matched to *n* = 1110 age‐ and residence‐matched controls that suffered from various nonmalignant disorders. This resembled a 1:6 matching ratio. All cancer cases and controls were identified during the same period and at the same institutions in order to minimize selection bias. All controls were admitted for medical conditions unrelated to stimulants (including alcohol and tobacco exposure). Controls that reported recent dietary modifications were excluded. Controls were admitted for the following medical conditions: abdominal hernia (*n* = 229, 20.6%), eye disorders (*n* = 246, 22.2%), urinary disorders (*n* = 125, 11.3%), injuries and trauma (*n* = 209, 18.8%), skin diseases (*n* = 88, 7.9%), appendicitis (*n* = 72, 6.5%), varicose veins (*n* = 43, 3.9%), hydatid cyst (*n* = 47, 4.2%), bone diseases (*n* = 28, 2.5%), and other medical disorders (*n* = 23, 2.1%).

Following Dupont's suggestions, a value of 0.2 was assumed as the correlation for the exposure rates between cases and controls.^40^ With a theoretical OR = 1.6 for disease in exposed/unexposed individuals, we needed at least 183 cases with six matched controls (1098) per case to reject the null hypothesis with a power = 0.80 and an α‐error = 0.05. The calculation was done with the online Epi R software (2.0 version, Melbourne, Australia, 2022).

### Questionnaire

The employed questionnaire covered sociodemographic and anthropometric variables. It has been described elsewhere in great detail.^41^ Moreover, the questionnaire inquired about potential history of substance usage (including tobacco and alcohol), cancer history in first to second degree relatives, and basic occupation titles. However, a very high fraction of patients labeled as “Retired” lacking another job title led us not to consider such data for the analysis. It also comprised a 64‐items food frequency questionnaire (FFQ) with good reproducibility which has been described earlier in detail.^41^

### Assessment of DAL

Two different markers were used to assess DAL in participants: Potential renal acid load (PRAL) and net endogenous acid production (NEAP).^42^, ^43^

PRAL was calculated as follows:

$$\text{PRAL}\;\left(\mathrm{mEq}/\mathrm{day}\right)=\left(0.49\times \text{total protein}\;\left[g/\mathrm{day}\right]\right)+\left(0.037\times \text{phosphorus}\left[\mathrm{mg}/\mathrm{day}\right]\right)-\left(0.021\times \text{potassium}\left[\mathrm{mg}/\mathrm{day}\right]\right)-\left(0.026\times \text{magnesium}\left[\mathrm{mg}/\mathrm{day}\right]\right)-\left(0.013\times \text{calcium}\left[\mathrm{mg}/\mathrm{day}\right]\right).$$

NEAP was estimated as follows:

$$\text{NEAP}\left(\mathrm{mEq}/\mathrm{day}\right)=\begin{array}{l} \left(54.5\times \text{protein}\left[g/\mathrm{day}\right]\right)/(0.0256\\ \times \text{potassium}\left[\mathrm{mg}/\mathrm{day}\right])–10.2. \end{array}$$

Negative PRAL scores reflect an alkaline‐forming potential, whereas positive scores indicate an acid‐forming potential. For NEAP scores, greater values indicate greater acid‐forming potentials.^39^ Both formulas are considered established tools and frequently used in clinical and epidemiological research. The first formula^43^ considers intestinal absorption rates of protein, potassium, phosphate, magnesium, and calcium. Moreover, it has been validated versus urinary pH in healthy individuals with good results.^43^ The second formula by Frassetto et al.^42^ takes into account the sulfuric acid production due to protein metabolism and the rate of bicarbonate production subsequent to the metabolization of intestinally absorbed potassium salts of organic acids. The pros and cons of both scores have been discussed in detail by Müller et al.^44^

### Statistical analysis

We used STATA software (Release 10, Stata Corp LP) to analyze our data. The majority of variables were treated as continuous, and categorization was done for analysis purposes only.

Descriptive statistics included frequencies for categorical variables and means (standard error in parenthesis) for continuous, normally‐distributed variables. Inferential statistics included odds ratios (ORs) and their corresponding 95% confidence intervals (95% CI) which were calculated using unconditional logistic regression. Potential confounders that were included in the multivariate logistic regression models included age, residence, family history of cancer in first and second‐degree relatives, body mass index, total energy intake, smoking history, mate intake, ethanol intake, and iron intake.

No participants were excluded as outliers for any dietary component. Heterogeneities in the stratified analyses were explored through likelihood‐ratio tests. Statistical results were regarded to be statistically significant when a two‐tailed *p‐*value was less than 0.05.

### Ethical approval and consent to participate

All participants gave verbal and written consent to participate in this study.

## RESULTS

Table 1 shows the distribution of cases and controls according to selected sociodemographic and habits variables. The study design yielded a distribution of age and residence (urban/rural status) with similar proportions. Education years were significantly less among cases. In addition, cases had a lower body mass index than controls and a higher smoking intensity than controls. No statistical differences were found concerning the family history of cancer rate.

**TABLE 1:** Selected sociodemographic characteristics and habits of the population under study (*n* = 1295). Distribution of cases and controls

Variables	Categories	Controls (n = 1110) %		Cases (*n* = 185) %		Global *p‐*value
Age groups	<40	6	0.5	1	0.5	
	40–49	91	8.2	16	8.6	
	50–59	201	18.1	37	20.0	
	60–69	383	34.5	63	34.1	
	70–79	345	31.1	52	28.1	
	≥ 80	84	7.6	16	8.7	0.96
Urban/rural status	Urban	822	74.0	131	70.8	
	Rural	288	26.0	54	29.2	0.35
Education years	None	161	14.5	36	19.5	
	1–4	476	42.9	90	48.6	
	≥5	473	42.6	59	31.9	0.02
FH of cancer in	No	773	69.6	137	74.0	
First and second degree	Yes	337	30.4	48	26.0	0.22
Body mass index	<18.50	20	1.8	7	3.8	
(kg/m^2^)	18.50–24.99	547	49.3	104	56.2	
	25.00–29.99	424	38.2	62	33.5	
	≥30.00	119	10.7	12	6.5	0.04
Smoking status	Nonsmoker	197	17.7	13	7.0	
	Ex‐smoker	406	36.6	57	30.8	
	Current smoker	507	45.7	115	62.2	<0.001
Smoking intensity	Nonsmoker	197	17.7	13	7.0	
(pack‐years)	0.1–28.6	339	30.6	33	17.9	
	28.7–55.0	315	28.4	60	32.4	
	≥55.1	259	23.3	79	42.7	<0.001

Abbreviation: FH of cancer, family history of cancer.

Table 2 presents selected nutritional variables, which were partially analyzed in categories and partially as mean values ± SD. Cancer cases had higher proportion of alcohols drinkers, higher “mate” intensity, and energy intake, but less tea and coffee consumption. In addition, cases showed higher mean intakes of red and processed meat, while their intake of white meat, fruits (all items significantly), and vegetables (marginally) were lower.

**TABLE 2:** Selected dietary features of the population under study (*n* = 1295). Distribution of cases and controls

Variables	Categories	Controls (*n* = 1110) %		Cases (*n* = 185) %		Global *p‐*value
Alcohol status	Never	281	25.3	30	16.2	
	Ex‐drinker	184	16.6	30	16.2	
	Current	645	58.1	125	67.6	0.02
“Mate” intensity	Nondrinkers	128	11.5	10	5.4	
(liters‐years)	0.1–54.9	499	45.0	82	44.3	
	≥55.0	483	43.5	93	50.3	0.03
Black tea intake	Never	843	76.0	179	96.8	
	Ever	267	24.0	6	3.2	<0.001
Coffee intake	Never	888	80.0	165	89.2	
	Ever	222	20.0	20	10.8	0.003
Dietary energy	≤1854	387	34.9	45	24.3	
(kcal/day)	1855–2354	369	33.2	64	34.6	
	≥2355	354	31.9	76	41.1	0.009

Food groups		Mean ± SD	Mean ± SD	*p*‐value
Red meat	Servings/year	391.9 ± 193.0	472.8 ± 220.9	<0.001
Processed meat	Servings/year	210.4 ± 189.9	247.1 ± 233.1	0.02
White meat	Servings/year	80.9 ± 72.8	60.3 ± 70.2	0.004
Total vegetables	Servings/year	561.3 ± 339.2	512.3 ± 371.9	0.07
Total fruits	Units/year	445.3 ± 332.0	310.9 ± 241.6	<0.001

Table 3 shows the mean values of both acid load scores (PRAL and NEAP) and their original components, and the latter expressed adjusted by 1000 kcal/day. Scores were significantly higher in cases than in controls. Regarding protein and phosphorus intakes, they were also statistically higher among cases. However, the intake of potassium, calcium, and magnesium showed no differences between controls and cases.

**TABLE 3:** Mean daily values ± standard errors of the acid load scores and their components. Stratification of items according to their animal/plant original source. Comparison between cancer cases and controls

Variable	Units	Controls (mean ± SE)	Cases (mean ± SE)	Diff. (*p*)
Total proteins	g/day	56.0 ± 0.6	61.1 ± 1.4	<0.001
Animal proteins	g/day	51.2 ± 0.6	56.6 ± 1.4	<0.001
Plant proteins	g/day	4.7 ± 0.1	4.6 ± 0.2	0.34
				
Total phosphorus	mg/day	803.9 ± 7.4	867.6 ± 17.7	0.001
Animal phosphorus	mg/day	478.0 ± 5.3	539.8 ± 14.7	<0.001
Plant phosphorus	mg/day	325.9 ± 4.0	327.7 ± 8.9	0.86
				
Total potassium	mg/day	1924.1 ± 18.5	1898.0 ± 42.6	0.59
Animal potassium	mg/day	688.5 ± 7.9	758.7 ± 22.1	0.001
Plant potassium	mg/day	1235.6 ± 15.0	1139.3 ± 34.4	0.01
				
Total magnesium	mg/day	182.5 ± 1.8	185.1 ± 4.2	0.59
Animal magnesium	mg/day	53.4 ± 0.6	59.8 ± 1.7	<0.001
Plant magnesium	mg/day	129.1 ± 1.6	125.3 ± 3.6	0.35
				
Total calcium	mg/day	608.7 ± 7.9	607.7 ± 15.7	0.96
Animal calcium	mg/day	350.5 ± 6.8	349.0 ± 13.1	0.93
Plant calcium	mg/day	258.2 ± 2.9	258.7 ± 7.2	0.95
		Mean ± SE	Mean ± SE	
PRAL score	mEq/day	4.10 ± 0.31	9.48 ± 0.79	<0.0001
NEAP score	mEq/day	53.29 ± 0.53	60.07 ± 1.24	<0.0001

Abbreviations: PRAL, potential renal acid load; NEAP, net endogenous acid production.

Table 4 shows the adjusted ORs for both acid load scores. Even the basic regression models (using the matching variables plus urban years) derived significant estimates: OR = 2.78, 95% CI: 1.82–4.24, ptrend <0.0001 for PRAL, and OR = 2.47, 95% CI: 1.63–3.76, ptrend <0.0001 for NEAP. In addition, the highest versus lowest tertile of PRAL derived significant adjusted estimates (OR = 2.28, 95% CI: 1.44–3.61, ptrend <0.0001). Similar results were found when analyzing the NEAP score: both risk and trend estimates were significant (OR = 2.17, 95% CI: 1.38–3.41, ptrend <0.0001). These scores were obtained using the most demanding regression model, which included age, residence, family history of cancer, body mass index, dietary energy, smoking status and intensity, filter use, “mate” status and intensity of intake, tea and coffee intake, and total iron intake. Finally, a regression model without the smoking variables was run, showing intermediate outcomes between the crude and the complete model, suggesting a role for the acid load per se, expressed in both scores.

**TABLE 4:** Crude and adjusted odds ratios (OR) of esophageal cancer for acid load scores (PRAL and NEAP). *p*‐values for their linear trends

	I		II		III		
	OR	95% CI	OR	95% CI	OR	95% CI	Trend (*p*)
PRAL (mEq/day)	**≤0.45**		**0.46–8.27**		**≥8.28**		
Model 1	1.00	**—**	**1.75**	1.12–2.74	**2.78**	1.82–4.24	<0.0001
Model 2	1.00	**—**	**1.74**	1.09–2.77	**2.28**	1.44–3.61	<0.0001
Model 3	1.00	**—**	**1.82**	1.15–2.87	**2.43**	1.56–3.79	<0.0001
NEAP (mEq/day)	**≤44.3**		**44.4–58.8**		**≥58.9**		
Model 1	1.00	**—**	**1.76**	1.14–2.73	**2.47**	1.63–3.76	<0.0001
Model 2	1.00	**—**	1.53	0.96–2.44	**2.17**	1.38–3.41	<0.0001
Model 3	1.00	**—**	1.57	0.99–2.50	**2.24**	1.43–3.51	<0.0001

*Note*: Regression models:

Model 1 = Adjusted by age (continuous), and residence (urban/rural).

Model 2 = Model 1 + family history of cancer in first and second degree (binary No/Yes) + body mass index (categorical, 4) + energy (continuous) + smoking status (categorical, 3) + smoking intensity (continuous) + filter use (continuous) + “mate” status + “mate” intensity (continuous) + tea intake (binary Never/Ever) + coffee intake (binary Never/Ever) + total iron (continuous).

Model 3 = Model 2 – smoking variables (status, intensity, filter).

Total iron = dietary iron/1000 kcal/day (in mg).

Abbreviations: PRAL, potential renal acid load; NEAP, net endogenous acid production.

## DISCUSSION

Our results demonstrate that higher acid load scores (both NEAP and PRAL) significantly increased the odds for ESCC. To the best of our knowledge, this is the first case‐control study to examine such an association in the English‐speaking scientific literature. Our findings are in line with two previous meta‐analyses that observed a significant association between higher DAL scores and the risk of cancer.^30^, ^31^

Several plausible pathophysiological mechanisms may explain the observed associations. It is noteworthy that a high DAL has been linked with various EC risk factors, including obesity and visceral fat,^45^ insulin resistance and type‐2 diabetes,^46^ decreased circulating adiponectin levels,^47^ increased IGF‐1 levels^48^ and obesity.^49^ Nevertheless, these factors might have a more substantial role in the EAC subtype.

Obesity significantly increases the risk of several chronic illnesses including cancer development.^50^ This also applies to EC cancer.^49^ Schlottmann et al.^49^ provided several possible explanations why EC is more common in obese individuals, including a higher prevalence of gastroesophageal reflux disease, linear associations between central adiposity and BE development; low levels of adiponectin, high serum leptin levels that potentially affect cell proliferation processes and changes in the esophageal microbiota due to unhealthy dietary habits that promote carcinogenesis. While the exact mechanisms are not fully understood, there is no doubt that obesity contributes to EC risk. Nevertheless, in our study, a higher proportion of overweight and obesity and a lower energy intake were found among control subjects. This point emphasizes that a higher alcohol intake among ESCC cases might have played a role in calorie intake and its proinflammatory and other known harmful properties.

In this context, a 2019 meta‐analysis by Farhangi et al.^51^ revealed that a high DAL content was associated with higher serum triglyceride concentrations and higher obesity prevalence. Several follow‐up studies in the last two years confirmed those findings and demonstrated that a high DAL is associated with obesity and abdominal obesity.^52^, ^53^ Therefore, although the associations between a high DAL and being overweight could potentially explain the associations between a high DAL and EAC, other factors warrant investigation because our findings on ESCC do not support overweight‐obesity as playing a role.

The consumption of a high DAL diet abundant in protein was found to significantly increase IGF‐1 levels in patients with type 2 diabetes.^54^ Long‐term consumption of high protein diets that increase DAL was found to be correlated with higher insulin growth factor (IGF‐1) serum levels and could also explain the increased odds for EC in individuals with a high DAL.^55^ Norat et al.^56^ examined the relationship of diet with serum insulin‐like growth factor‐I (IGF‐I) in more than 2000 women and reported that IGF‐I levels were positively related to protein intake (*p*[trend] < 0.001), milk intake (*p*[trend] = 0.007) and phosphorus intake (*p*[trend] < 0.001). An inverse relationship was found for vegetables (*p*[trend] = 0.02) and beta‐carotene (*p*[trend] = 0.02). Findings by Crowe et al.^57^ essentially confirmed those results. If we look at how DAL scores are estimated, it is conceivable that the association between DAL and IGF‐1 could explain our observations and estimated OR concerning ESCC, despite the role played by tobacco smoking, alcohol drinking, and very hot “mate” drinking, which are already reported risk factors for ESCC.^8^, ^9^, ^10^, ^11^, ^12^

Discussing DAL calculations, it is also essential to emphasize that PRAL‐lowering foods (fruits, vegetables and legumes) are frequently associated with reduced odds for developing EC.^58^, ^59^ Intake of rosacea (apples, peaches, nectarines, plums, pears and strawberries) and rutaceae (citrus fruits) are particularly associated with protective associations for developing ESCC.^60^ Thus, our findings are indirectly in line with previous studies.

Nevertheless, this study has an explorational character and is – to the best of our knowledge ‐ the first analysis to investigate potential associations between EC, particularly ESCC, and DAL. Additional trials are thus warranted to confirm our findings.

Our study has multiple strengths and weaknesses that warrant a detailed discussion. As for the strengths, all the interviews with participants were done face‐to‐face (excluding proxy interviews) by the same interviewers at the same institutions to reduce potential selection bias. In addition, the selected population sample was comprehensive from the viewpoint of country areas and socioeconomic subsets. The low attrition rate of identified cases and controls (rates ~3%), favored by the interview performed during the hospital stay, limited possible selection bias. Another strength is the exclusion of individuals that reported previous (major) dietary modifications.

As for the weaknesses, the study included a detailed but nonvalidated FFQ. However, as reported earlier, the FFQ was shown to be satisfactorily reproducible. Although our investigation dates back to the early 2000s, the general dietary habits have not changed a lot in Uruguay,^61^ and recent investigations demonstrated the same heavily meat‐based pattern. We must also acknowledge that our analysis did not include EC‐related confounders, such as occupational and home exposure to smoking and other kinds of pollution (polycyclic aromatic hydrocarbons, N‐nitroso compounds, acetaldehyde, and fumonisins) that potentially play a role in EC development.^62^ A significant fraction of patients were assigned to a category of “Retired” without mentioning the previous job title(s): this was the main reason for not considering those data in the analysis. Finally, the study was designed only to be performed on men, an additional limitation since esophageal cancer was not among our country's most frequent female cancers before the study began. This now represents a lack of information for the desirable comparison between sexes.

In conclusion, we present evidence for a potential significant association between elevated DAL scores and increased odds for ESCC. In both cases, findings were supported by complex regression models adjusting for multiple confounders. This is the first study to suggest that an acidogenic diet high in animal protein may have contributed to an increased EC risk in the examined population. Our results are in line with several previous epidemiological studies on dietary patterns and EC risk. Nevertheless, additional investigations are warranted to confirm our findings, since there are two distinct subtypes of EC and the present study was focused on one of both.

## AUTHOR CONTRIBUTION

ALR participated in the original idea, design, data processing, statistical analyses, text redaction, and general supervision; MAS collaborated in the text redaction, draft supervision, graphic design, language checking, and general supervision; WML collaborated in the text supervision, biochemical and molecular supervision, and final draft supervision; JMC collaborated in tables design and the text supervision.

## CONFLICT OF INTEREST

The authors declare that they have no known competing financial interests or personal relationships that could have appeared to influence the work reported in this paper.
